# Genetically modified insects as a public health tool: discussing the different bio-objectification within genetic strategies

**DOI:** 10.3325/cmj.2012.53.635

**Published:** 2012-12

**Authors:** Luísa Reis-Castro

**Affiliations:** Institute for Social and Cultural Anthropology, Martin-Luther-University of Halle-Wittenberg, Germany

The management of infectious disease vectors includes a wide range of strategies to control the population of species that carry and transmit infectious pathogens. Although vectors can include a variety of animals and microorganisms, this article addresses particularly mosquitoes that act as potential disease carriers. Regarding concerns from a social science and public heath perspective, the management of these disease vectors can reflect quite different priorities and strategies, which have significantly different public policies implications.

Vector control presupposes that the key manner to tackle a disease is by controlling the population that transmits the pathogen. Genetic strategies are a relatively recent approach, in which vectors are genetically engineered so they themselves can be adopted in strategies for public health. DNA with the desired genes is inserted at a specific moment into the eggs of these insects, in order to alter their behavior and/or biology. The genetic manipulation of these creatures challenges the boundaries between natural and artificial, and transforms organisms into objects to be used as a public health tool. This process alters the identity of these insects and can be defined as bio-objectification, as already discussed in other articles within this journal (1,2). Beisel and Boëte examined how the use of GM mosquitoes in tackling malaria could significantly transform the way disease control strategies are organized (3). In short, it transforms the disease vector into another entity that the authors define as a “flying public health tool.” This change has significant implications for public health knowledge and practices. The same argument can be made for genetic strategies dealing with other mosquito-borne diseases. The genetically engineered insects embody the management of an infectious disease in their very genome. The modification transforms the mosquito from an entity that causes problems to one that brings the answer: it becomes a tool to solve the very problem it causes.

Genetic strategies for vector control are usually divided into to two distinct groups: the first is *population suppression, containment, or eradication,* while the second is *population transformation or replacement*. In this article I describe these two approaches, arguing that once outside the laboratory they involve two different bio-objectification processes and result in very distinct modes of governance *through* and *of* these GM insects (4,5).

## Population suppression, containment, or eradication

The approach of *population suppression, containment, or eradication* targets the reduction or even elimination of specific insect species by developing genes that are (conditionally) lethal or make the insect unable to reproduce. There are a variety of possible genes with different workings currently being researched in laboratories around the world. However, one system within this approach is already in GM insects flying outside the walls of the laboratory. Mosquitoes containing the Release of Insects Carrying a Dominant Lethal (RIDL) system have been set free in Cayman Islands, Malaysia, and Brazil. Since these are the first and only releases it is of interest to discuss this genetic strategy in more detail.

The core of the RIDL system is the tetracycline-repressible expression system (6). In brief, in this system the tetracycline acts as a chemical switch: the insect is conditioned to only survive to adulthood if in the presence of this antibiotic. These insects can be mass reared since inside the laboratory the growing larvae can be fed tetracycline. Mass production of these insects is required for the large and continuous releases required when adopting this (bio)technological strategy. The population is to be reduced by releasing the genetically modified mosquitoes containing the conditionally lethal gene. The repeated and constant releases are required to maintain the GM population in the environment. Eradication is usually difficult to accomplish. In case it is not achieved, when releases are stopped, the non-GM mosquito population will increase again (7).

The RIDL (bio)technology was developed by Oxitec (a spin-off from Oxford University) and adopted to tackle dengue fever disease. Oxitec scientists genetically modified the *Aedes aegypti*, a vector for dengue fever disease, creating the patented RIDL product of the strain *Ae. aegypti* OX513A.

Since only *Ae. aegypti* females can act as vectors, Oxitec and its collaborators plan to release OX513A males to mate with wild females. Their progeny would be heterozygous for the lethal gene and still express it – that is, the heterozygous offspring needs tetracycline to achieve adulthood.

The heterozygotes survive through the larvae phase but should not reach adulthood, what is defined as late-lethality. However, some of these heterozygous do become adult mosquitoes. In laboratory experiments, a 3%-4% survival rate to adulthood is acknowledged (8). A confidential paper by Oxitec, made public by the non-governmental organization GeneWatch UK, showed a survival rate of even 15% among the heterozygous; this was apparently because they were being fed cat food containing tetracycline (9,10). In the case of large-scale production even very small percentage could result in considerable side-effects.

The first field trial of the RILD GM mosquitoes took place in the Cayman Islands, a British Overseas Territory, and it was conducted in collaboration with the Cayman Islands’ Mosquito Research and Control Unit (MRCU). From late 2009 to October 2010, three million OX513A mosquitoes were set free on the island (11). The second release happened in Malaysia and was carried out by the local Institute for Medical Research (IMR) where from December 21, 2010 to January 5, 2011 six thousand GM mosquitoes were set free into the environment (12). The third experiment is being conducted in Brazil under the coordination of the University of Săo Paulo and it is the only location where releases are still occurring. It is also the only release of large scale with more than fifteen million insects released between 2011 and 2012 (13). Moreover, the genetic strategy for dengue control seems set to continue in Brazil, with the construction of a new “biofactory:” the Production Unit for Transgenic *Aedes* (UPAT) which can mass rear four million mosquitoes per week (14).

## Population transformation or replacement

When considering strategies geared toward *population transformation or replacement,* the aim is not to eliminate the vector but to create a substitution, avoiding the emergence of an empty ecological niche. This is done through a genetic modification that either reduces or blocks the insect’s ability to transmit a disease. The goal here is to change a population of vectors into a population of non-vectors.

Such approach always involves the development of a system with two different types of genes, which are researched independently. The first are *refractory genes* and they modify the insects so that, in case the mechanism is fully efficient, they can no longer serve as disease vectors – ie, they can no longer transmit the pathogen. Besides that, they also require the development of *gene drive systems* that favor the spread of refractoriness in the population. They support the fixation of the allele of interest into the wild population in order to transform it (15). It requires releases of sufficient numbers of genetically engineered organisms to pass a threshold to ensure that GM individuals will replace the entire wild type population. In theory, once this threshold has been passed, the transgene is self-sustaining and releases would need to occur only once or a few times (7). Nonetheless, the strategies within this approach are still in development at laboratories and these relations with the environment are somewhat speculative (16).

## Differences in the “field:” Distinct governances *through* and *of* these bio-objects

GM insects from both approaches have been genetically manipulated in order to transform their biological forms. They emerge as clear examples of *bio-objects*, as they are living organisms transformed through scientific labor into something that can be (mass) produced, leveraged, circulated, regulated, and adopted as a public health tool (2). These bio-objects are designed to increase control over a mosquito-borne disease and improve *human* life – sometimes at the expense of other species.

Regarding the laboratory identity, GM insects from the two different approaches do not differ much, all being imbued with hopes and expectations of being a solution for tackling mosquito-borne diseases, and at the same time triggering a series of concerns and fears. However, once outside the laboratory and as (potentially) part of public policies, their bio-objectification results in very distinct modes of governance *through* and *of* these GM insects.

The governance of a health problem such as dengue and other mosquito-borne disease *through population suppression* demands constant mass rearing and constant releases of these insects. As noted, as soon as the releases are stopped or paused, the size of the non-GM population tends to increase again. This demands a large infrastructure for the constant “production” of these bio-objects. For example, in an interview the coordinator of the Brazilian project mentioned that during a week they were forced to reduce the number of released GM mosquitoes, due to shortage of sufficient blood to mass rear the necessary amount (M. Capurro, personal communication, September 17, 2012). This need for continuous releases results in a dependence and lock-in concerning the production of this living technology as a public health policy tool. This is an important characteristic to take into consideration, especially in the case of patented technologies and commercial interest.

The governance of a mosquito-borne disease *through* a GM insect would happen in a very different manner in the case of *population transformation*. If it successfully transforms the population, it would be done through a one-time release or a few releases and thus would require a much smaller infrastructure for “production.” Mosquitoes within this latter approach are designed to be self-sustaining; however, their functioning can fail due to phenomenon called “gene silencing,” which takes place when copies of introduced genes are inactivated. The reason why genes are silenced or completely removed is still somehow unclear, but scientists speculate it is a “defense mechanism intended to prevent genetic damage” (16).

Regarding the governance *of* the GM insects within these two approaches also two distinct regulatory frameworks are required. In the case of population suppression, “the novel trait is expected to disappear more-or-less rapidly from the environment after releases” (17). Hence, after a few generations the genetically modified trait should die out if releases cease, resulting in a much easier reversibility but also possible re-creation of the public health problem. Within the population transformation strategies, reversibility is a much more complicated matter, as they have been engineered to maintain themselves within the wild population. One option would be to release wild type (non-GM) mosquitoes to remove modified alleles from the population (18). In case the refractoriness gene is silenced, the mosquito would once again become a vector and could also re-create the public health problem. Within population transformation strategies another hybridity has emerged: the wild population has been *transformed* into a GM one, challenging and re-drawing once again the boundaries between natural and artificial, wild and laboratory-designed.

Within both approaches, an important issue to be considered in the governance of these disease control strategies is who should be responsible for overseeing these bio-objects and accountable for the implications of their use. For example, the *location of responsibility* (5) in the three releases conducted so far is unclear: if it is at Oxitec, at the local institutes conducting the releases, or at the policy makers and regulators of the county who have assessed and authorized the experiments.

The distinct ways of modifying the insect to combat mosquito-borne diseases reflect very different conditions and configurations of a public health policy. Within the population suppression group, it favors a bio-objectification process more in tune with commercial interests, since it implicates a continuous “production” and “consumption” of these GM mosquitoes. In contrast, the population transformation involves a bio-objectification process that might be less commercially attractive. The argument above indicates that public health and, crucially, the resources needed for it in low-income countries, might be better served by the strategies within the second approach. Nevertheless, the more complex conditions for reversibility that comes with population transformation approach could implicate challenges and higher risks for humans and the environment.

In conclusion, these approaches reflect significantly different priorities and implications within public health policies. Notwithstanding these differences, it should not be taken for granted they still share the standpoint that focuses on vector control as the key to tackle mosquito-borne disease, possibly overlooking other critical variables such as quality of primary health care (19,20).
